# Sex Differences in Abnormal Intrinsic Functional Connectivity After Acute Mild Traumatic Brain Injury

**DOI:** 10.3389/fncir.2018.00107

**Published:** 2018-11-29

**Authors:** Shan Wang, Liuxun Hu, Jieli Cao, Wenmin Huang, Chuanzhu Sun, Dongdong Zheng, Zhuonan Wang, Shuoqiu Gan, Xuan Niu, Chenghui Gu, Guanghui Bai, Limei Ye, Danbin Zhang, Nu Zhang, Bo Yin, Ming Zhang, Lijun Bai

**Affiliations:** ^1^The Key Laboratory of Biomedical Information Engineering, Ministry of Education, Department of Biomedical Engineering, School of Life Science and Technology, Xi’an Jiaotong University, Xi’an, China; ^2^Department of Neurosurgery, The Second Affiliated Hospital and Yuying Children’s Hospital of Wenzhou Medical University, Wenzhou, China; ^3^Department of Medical Imaging, The First Affiliated Hospital of Xi’an Jiaotong University, Xi’an, China; ^4^Department of Radiology, The Second Affiliated Hospital and Yuying Children’s Hospital of Wenzhou Medical University, Wenzhou, China

**Keywords:** mild traumatic brain injury, sex difference, rs-fMRI, functional connectivity, independent component analysis

## Abstract

Mild traumatic brain injury (TBI) is considered to induce abnormal intrinsic functional connectivity within resting-state networks (RSNs). The objective of this study was to estimate the role of sex in intrinsic functional connectivity after acute mild TBI. We recruited a cohort of 54 patients (27 males and 27 females with mild TBI within 7 days post-injury) from the emergency department (ED) and 34 age-, education-matched healthy controls (HCs; 17 males and 17 females). On the clinical scales, there were no statistically significant differences between males and females in either control group or mild TBI group. To detect whether there was abnormal sex difference on functional connectivity in RSNs, we performed independent component analysis (ICA) and a dual regression approach to investigate the between-subject voxel-wise comparisons of functional connectivity within seven selected RSNs. Compared to female patients, male patients showed increased intrinsic functional connectivity in motor network, ventral stream network, executive function network, cerebellum network and decreased connectivity in visual network. Further analysis demonstrated a positive correlation between the functional connectivity in executive function network and insomnia severity index (ISI) scores in male patients (*r* = 0.515, *P* = 0.006). The abnormality of the functional connectivity of RSNs in acute mild TBI showed the possibility of brain recombination after trauma, mainly concerning male-specific.

## Introduction

Traumatic brain injury (TBI) is a substantial public health problem, and can accelerate the ageing process, leading to long-term structural and functional alterations to the brain (Benedictus et al., 2010; Cole et al., 2015). About 90% of TBI is classified as mild (Vos et al., 2002). It is worthy of attentions that one-quarter of mild TBI patients have post-concussive symptoms or other cognitive disorders (Bazarian et al., 2010). However, the heterogeneity of the injuries and the variability of cognitive symptoms make it problematic for management (Jenkins et al., 2016). Several factors are associated with poor outcomes after mild TBI, of which, the most controversial is sex (Bazarian et al., 2010). In female group, sex steroids have been tested to demonstrate neuroprotective effects in severe TBI (Fakhran et al., 2014). Nevertheless, the effect of sex on outcome is still unclear after mild TBI. Understanding sex differences of brain injury mechanism after mild TBI may enhance the future diagnostic work-up in patients and lead to separate management strategies for patients with different sexes.

Previous studies about sex differences in cognitive outcome after mild TBI revealed controversial and interesting results. Controlled animal experiments have shown better cognitive outcomes among females after mild TBI (Bramlett and Dietrich, 2001; O’Connor et al., 2003; Bazarian et al., 2010). Evidence from a human study also finds that women have superior executive functioning when compared with men after acute TBI (Niemeier et al., 2014). Other studies show that women outperform men in the tasks of verbal memory and learning measures following TBI (Farace and Alves, 2000; Covassin et al., 2012). These results indicate that female sex is somehow neuroprotective. While, multiple observational studies in humans have demonstrated that females present worse outcomes following concussion compared with males (Broshek et al., 2005; Bay et al., 2009; Covassin et al., 2013; Hsu et al., 2015; Cancelliere et al., 2016). Specifically, females report more post-concussive symptoms with greater severity compared to males (Broshek et al., 2005; Bay et al., 2009; Covassin et al., 2012). Indeed, these observation studies may be confounded by many factors, especially by sociological pressures on male athletes, including the greater societal stigma with symptom reporting, resulting in underreporting by males (Fakhran et al., 2014). Therefore, more objective measurements, such as neuroimaging indices, are crucial in avoiding such bias.

The evaluation of resting-state functional connectivity is an appealing approach to assess activity differences among sexes (Filippi et al., 2013). A recent research has revealed that global connectivity was stronger in female network than in males with posttraumatic stress disorder (PTSD; Cao et al., 2018). However, resting-state fMRI studies in mild TBI focus on connectivity mainly in a limited number of predefined regions-of-interest (ROIs), not fully exploring large-scale brain functional connectivity (Mayer et al., 2011; Slobounov et al., 2011; Shumskaya et al., 2012). Though substantial evidence supports models of TBI as a condition characterized by altered brain connectivity, sex-related differences in functional connectivity are still less clear. Understanding the effects of sex difference after mild TBI on brain function and behavior is likely to require a widespread investigation on brain network connectivity. Indeed, several studies have characterized the resting-state networks (RSNs) using independent component analysis (ICA; Damoiseaux et al., 2006; De Luca et al., 2006). As declared by a study performed on a very large sample of healthy participants, these networks have a high reproducibility (Biswal et al., 2010). The assessment of RSNs allows us to evaluate the intrinsic functional connectome of the human brain among sexes (Filippi et al., 2013).

Most studies investigating functional connectivity have involved patients with moderate-to-severe TBI, or during the chronic stage of recovery (Caeyenberghs et al., 2012, 2013, 2014; Shumskaya et al., 2012). Importantly, reliable and valid indices of acute injury, which can elucidate underlying neuroanatomical injury mechanisms or be predictive for longer-term outcomes, are lacking in studies of mild TBI (Yuan et al., 2015). Thus, in the current study, we aimed to investigate the sex differences on whole-brain functional connectivity at the network level from a cohort of acute mild TBI patients, not biased by *a priori* region selection.

## Materials and Methods

### Participants

A total of 61 patients with acute mild TBI within 7 days post-injury were recruited from the emergency department (ED) of a local hospital, between August 2016 and July 2017. All consecutively patients with non-contrast head CT due to acute head trauma enrolling from the local ED formed the initial population. Inclusion criteria for all mild TBI patient were based on the World Health Organization’s Collaborating Centre for Neurotrauma Task Force (Holm et al., 2005): (i) Glasgow Coma Scale (GCS) score of 13–15 on presentation to the ED; (ii) one or more/any of the following: loss of consciousness (LOC) for less than 30 min, posttraumatic amnesia (PTA) for 24 or less hours, and/or other transient neurological abnormalities such as focal signs, seizure, and intracranial lesion not requiring surgery; and (iii) were aged 18 years or older. Mild TBI patients were excluded for: (i) a history of a previous brain injury, neurological disease, long-standing psychiatric condition, or concurrent substance or alcohol abuse; (ii) a structural abnormality on neuroimaging (CT and MRI); (iii) intubation and/or presence of a skull fracture and administration of sedatives; (iv) the manifestation of mild TBI due to medications by other injuries (e.g., systemic injuries, facial injuries, or spinal cord injury); (v) other problems (e.g., psychological trauma, language barrier, or coexisting medical conditions); and (vi) caused by penetrating craniocerebral injury. Among these patients, seven were excluded, five of whom had MRI contraindications, and two were left handedness. At last, 54 patients (27 males) were enrolled. In addition, 34 sex-, age- and education-matched healthy controls (HCs; 17 males) without neurologic impairment or psychiatric disorders participated in the study. All participants were right-handed according to the Edinburgh Handedness Inventory. All the subjects gave written, informed consent in person approved by a local institutional review board; the research procedures were approved by the Ethical Committee of The Second Affiliated Hospital of Wenzhou Medical University and conducted in accordance with the Declaration of Helsinki.

### Clinical Assessment

Clinical assessments were performed within 48 h of MR imaging for all the participants. Neuropsychological tests included: (a) WAIS-III Digit Symbol Coding (DSC) to examine motor skill and memory; (b) Verbal Fluency Test to evaluate verbal fluency including language ability, executive function and semantic memory (Troyer et al., 1997; Kreiner and Ryan, 2001). Self-assessment symptom questionnaires included: the Rivermead Post-Concussion Symptom Questionnaire (RPCS), Insomnia Severity Index (ISI; King et al., 1995; Bastien et al., 2001).

### Image Acquisition

A non-contrast CT scan was performed on all consecutive patients following acute head injury with a 64-row CT scanner (GE, Lightspeed VCT). The MRI scans were acquired with the use of 3.0 T MRI scanner (GE 750). A custom-built head holder was used to prevent head movements. All participants were instructed to remain in a relaxed state without engaging in cognitive or motor activity and to keep their eyes closed. Alertness during the scan was confirmed immediately afterward. The MRI protocol involved the high-resolution T1-weighted 3D MPRAGE sequence (echo time (TE) = 3.17 ms, repetition time (TR) = 8.15 ms, flip angle = 9°, slice thickness = 1 mm, field of view (FOV) = 256 mm × 256 mm, matrix size = 256 × 256), single-shot, gradient-recalled echo planar imaging (EPI) sequence with 54 slices covering the whole brain (TR = 2,000 ms, TE = 30 ms, slice thickness = 3 mm, flip angle = 90°, FOV = 216 mm × 216 mm, matrix size = 64 × 64, voxel size = 3 mm × 3 mm × 3 mm), axial FLAIR (TR = 9,000 ms, TE = 95 ms, flip angle = 150°, thickness = 5 mm, slices = 20, FOV = 240 mm × 240 mm, matrix size = 173 × 256), axial susceptibility weighted imaging (SWI; TR = 37.8 ms, TE = 25 ms, flip angle = 15°, thickness = 2 mm, slices = 70, FOV = 230 mm × 230 mm, matrix size = 512 × 512), axial FLAIR (TR = 9,000 ms, TE = 95 ms, flip angle = 150°, thickness = 5 mm, slices = 20, FOV = 240 mm × 240 mm, matrix size = 173 × 256).

The presence of focal lesions and cerebral microbleeds was determined by an experienced clinical neuroradiologists (with 10 years’ experience) who assessed multiple modalities of neuroimaging data acquired at baseline (T1-weighted, SWI, FLAIR) for all subjects in random sequence, blind to clinical information and group membership (patient or control).

### Preprocessing of Resting State Data

Image preprocessing was accomplished using the FSL software package (Smith et al., 2004). First, the first 10 volumes of resting-state data were removed to allow for steady state equilibrium. Data preprocessing included the slice-timing, head-motion correction, normalization, spatially smoothing with a 6-mm full width at half maximum, linear trend removal, and band-pass filtering (0.01–0.08 Hz). Motion correction was performed by realigning fMRI time-series using a six-parameter rigid-body spatial transformation (Friston et al., 1995). In the normalization step, all BOLD data were aligned to their corresponding T1-weighted images, and normalized BOLD images were created by applying the transformation of T1-weigthed images to the ICBM152 template. Spurious variances (head motion, ventricular and white matter signal and the derivatives of each of these signals) were removed by multiple linear regression analysis.

Data analysis was performed using the FMRIB Software Library (FSL; FMRIB Software Library). Head motion in the resting state data was corrected using multi-resolution rigid body co-registration of volumes, as implemented in the MCFLIRT software (Jenkinson et al., 2002). Brain extraction was carried out in the BET software for motion-corrected BOLD volumes with optimization of the deforming smooth surface model, as implemented (Smith, 2002). Rigid body registration as implemented in the FLIRT software was used to co-register fMRI volumes to 3D MPRAGE (brain-extracted) volumes of the corresponding subjects and subsequently the 3D MPRAGE volumes to the MNI152 standard space (Jenkinson et al., 2002). The images were smoothed with a 6-mm full width at half-maximum (FWHM) Gaussian kernel.

### Resting State Networks

Following the preprocessing, Multivariate Exploratory Linear Optimized Decomposition into Independent Components (MELODIC) tool within FSL was used to perform spatial group-ICA using multisession temporal concatenation. Datasets were temporally concatenated across all participants to create a single 4-dimensional dataset as input for MELODIC, to produce 25 independent component maps (IC maps) representing average resting state brain networks (RSNs).

The intrinsic functional connectivity was the connectivity among various regions within a RSN (Kumar et al., 2018). A dual regression approach was used to perform the between-subject analysis by voxel-wise comparisons of resting functional connectivity (Jenkinson et al., 2002; Nichols and Holmes, 2002; Beckmann and Smith, 2005; Cole et al., 2010; Kumar et al., 2018). We accomplished this procedure as follow. First, we used all group ICA spatial maps as spatial regressors against the preprocessed individual subject’s fMRI data, which produced subject-specific time courses for each group ICA component. Then, these time courses were variance-normalized and linearly regressed for the subject’s fMRI dataset. Individual spatial maps of each group ICA component were provided by the regression. Finally, we merged these individual spatial maps across subjects into single 4-dimensional files per ICA component. The voxel-wise group differences in intrinsic network functional connectivity between male and female patients were carried out using nonparametric permutation testing (5,000 permutations per contrast for each ICA component) in FSL (Kumar et al., 2018). Threshold-free cluster enhancement (TFCE) was used to control the multiple comparisons. The significance threshold was set to *P* < 0.05, Family-Wise Error (FWE) corrected. The results represented the group differences in functional connectivity for all RSNs.

### Region of Interest Analysis

Region-of-Interest (ROI) analysis was then applied based on the regions showing significant differences on functional connectivity between male and female patients. Such analysis would provide us the ability to determine the size and location of the clusters, which showed significant differences between male and female patients. A single ROI mask was created according to the location and size of clusters in the specific RSN. Strengths of functional connectivity of patients and HCs, were then extracted in an automated fashion by using the ROI mask along the individual spatial maps.

### Additional Statistical Analysis

Statistical analyses were performed in SPSS 20.0. For each continuous variable, the normal distribution was measured by the Shapiro-Wilk test. The independent two-sample *t*-test and the Mann-Whitney test were used to compare group differences based on data normality, respectively. A Kruskal-Wallis test was conducted to assess the differences in age, education level and neuropsychologic test results in four groups (i.e., control male, control female, mild TBI male and mild TBI female). Chi-square analyses were applied to assess categorical variables. ROI-analysis among four groups were subjected to the univariate analysis of variance (ANOVA). Correlation analysis was also conducted between functional connectivity and clinical symptoms by using Pearson correlation coefficients.

## Results

### Participant Characteristics

During the study, we employed 54 patients with mild TBI, all of which were recruited from the ED of the local Level-1 emergency center. None of patients were with visible contusion lesions using conventional neuroimaging techniques and exhibited cerebral microbleeds on SWI. Thirty-four (17 males) control subjects were included. No significant difference was seen between the HCs and patients with mild TBI in regard to age, sex and education level (*P* > 0.1).

For all the patients, no significant contusion or cerebral hemorrhage was found. The major mechanism of trauma was a motor vehicle collision injury [13 of 27 male patients (48.2%), 15 of 27 female patients (55.6%)], followed by assault [9 of 27 male patients (33.3%), 7 of 27 female patients (25.9%)], and fall was the last [5 of 27 male patients (18.5%), 5 of 27 female patients (18.5%)]. No significant differences in age and education level were found between male and female patients with mild TBI (*P* > 0.05).

Both female and male patients with mild TBI displayed deficits on all of the clinical assessments compared with their control counterparts. For both the male and female subjects, there were significant differences between patients and controls in the Rivermead Post-Concussion Symptom Questionnaire (*P* < 0.001), WAIS-III DSC score (*P* < 0.05), Verbal Fluency Test (*P* < 0.05), ISI (*P* < 0.001; Table 1). No significant sex differences were found for all assessments, neither in mild TBI nor HC groups (Table 2).

**Table 1:** Demographic and behavioral statistics for male and female participants.

		Male participants			Female participants		
Characteristic	Mild TBI group (*n* = 27)	Control group (*n* = 17)	*P* value (Cohen’s *d*)	Mild TBI group (*n* = 27)	Control group (*n* = 17)	*P* value (Cohen’s *d*)
**Demographic**						
Age (years)	35.4 ± 9.7 (19–54)	32.8 ± 10.1 (22–53)	0.394(0.272)	35.6 ± 9.4 (21–54)	33.1 ± 10.7 (20–54)	0.421 (0.254)
Education (years)	9.4 ± 3.5 (3–15)	10.7 ± 5.1 (1–17)	0.322 (−0.305)	9.5 ± 4.3 (0–16)	11.1 ± 4.8 (0–17)	0.270(−0.351)
**Clinical assessments**							
RPCS	8.4 ± 5.8 (1–23)	1.3 ± 1.8 (0–7)	< 0.001 (1.669)	11.4 ± 6.4 (3–25)	2.4 ± 2.5 (0–9)	< 0.001 (1.915)
DSC	34.7 ± 13.7 (14–60)	50.7 ± 15.4 (14–60)	0.001 (−1.130)	36.7 ± 14.1 (9–60)	48.8 ± 15.7 (16–60)	0.011 (−0.833)
VF	17.1 ± 4.8 (9–27)	20.8 ± 6.7 (9–32)	0.042 (−0.641)	15.6 ± 4.8 (9–26)	19.1 ± 6.3 (8–31)	0.043 (−0.642)
ISI	5.9 ± 4.5 (0–17)	1.2 ± 2.6 (0–11)	< 0.001 (1.307)	8.8 ± 7.1 (1–24)	1.8 ± 2.2 (0–8)	< 0.001 (1.357)

*Notes: DSC, WAIS-III Digit Symbol Coding score; VF, Verbal Fluency Test; RPCS, the Rivermead Post-Concussion Symptom Questionnaire; ISI, Insomnia Severity Index; In 2nd, 3rd, 5th, 6th column, numbers in parentheses are ranges. In 4th, 7th column, numbers in parentheses are for the Cohen’s *d* effect size. *P* < 0.05 was considered statistically significant in all the tests*.

**Table 2:** Demographic and behavioral statistics for patients and control subjects.

	Patients with mild TBI			Control subjects		
Characteristic	Male (*n* = 27)	Female (*n* = 27)	*P* value (Cohen’s *d*)	Male (*n* = 27)	Female (*n* = 27)	*P* value (Cohen’s *d*)
**Demographic**	
Age (years)	35.4 ± 9.7 (19–54)	35.6 ± 9.4 (21–54)	0.996 (−0.012)	32.8 ± 10.1 (22–53)	33.1 ± 10.7 (20–54)	0.948 (−0.023)
Education (years)	9.4 ± 3.5 (3–15)	9.5 ± 4.3 (0–16)	0.945 (−0.019)	10.7 ± 5.1 (1–17)	11.1 ± 4.8 (0–17)	0.837 (−0.073)
**Clinical assessments**
RPCS	8.4 ± 5.8 (1–23)	11.4 ± 6.4 (3–25)	0.073 (−0.507)	1.3 ± 1.8 (0–7)	2.4 ± 2.5 (0–9)	0.166 (−0.502)
DSC	34.7 ± 13.7 (14–60)	36.7 ± 14.1 (9–60)	0.593 (−0.149)	50.7 ± 15.4 (14–60)	48.8 ± 15.7 (16–60)	0.726 (0.125)
VF	17.1 ± 4.8 (9–27)	15.6 ± 4.8 (9–26)	0.242 (0.328)	20.8 ± 6.7 (9–32)	19.1 ± 6.3 (8–31)	0.450 (0.270)
ISI	5.9 ± 4.5 (0–17)	8.8 ± 7.1 (1–24)	0.072 (−0.509)	1.2 ± 2.6 (0–11)	1.8 ± 2.2 (0–8)	0.441 (−0.276)

*Notes: DSC, WAIS-III Digit Symbol Coding score; VF, Verbal Fluency Test; RPCS, the Rivermead Post-Concussion Symptom Questionnaire; ISI, Insomnia Severity Index; In 2nd, 3rd, 5th, 6th column, numbers in parentheses are ranges. In 4th, 7th column, numbers in parentheses are for the Cohen’s *d* effect size. *P* < 0.05 was considered statistically significant in all the tests*.

### Resting-State Networks

**Figure 1:**
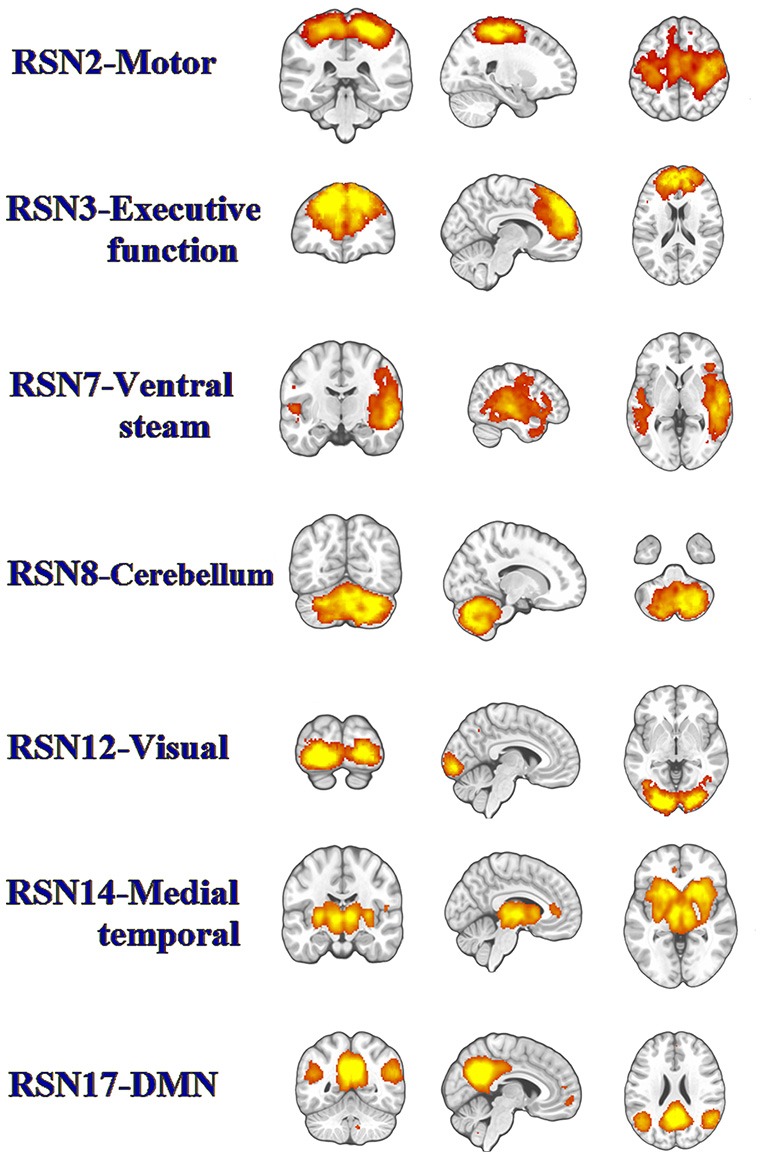
The panel represents functionally relevant resting-state networks (RSNs) from the group independent component analysis (ICA) analysis of temporally concatenated datasets from both patients with mild traumatic brain injury (TBI) and control subjects. The left side of the brain corresponds to the left side in the image.

Twenty-five components were computed in the entire subject group by the ICA. Based on visual inspection of the spatial map (biologic plausibility and comparability with previously reported RSNs), we sorted the components into functionally relevant RSNs and artifactual components related to physiologic/scanner noise and head motion. Seven components closely coincided with prior reports (Figure 1; Shumskaya et al., 2012). RSN 2 corresponded to the motor network. The visual processing network was represented in the RSN 12. We also found two RSNs involved in high-order cognitive functions: the executive function network (RSN 3) and default mode network (RSN 17). RNS14 was medial temporal which located in the temporal lobe. RNS7 corresponded to the ventral stream. We identified another component that was rarely reported or examined thoroughly, namely the cerebellum (RSN 8).

**Figure 2:**
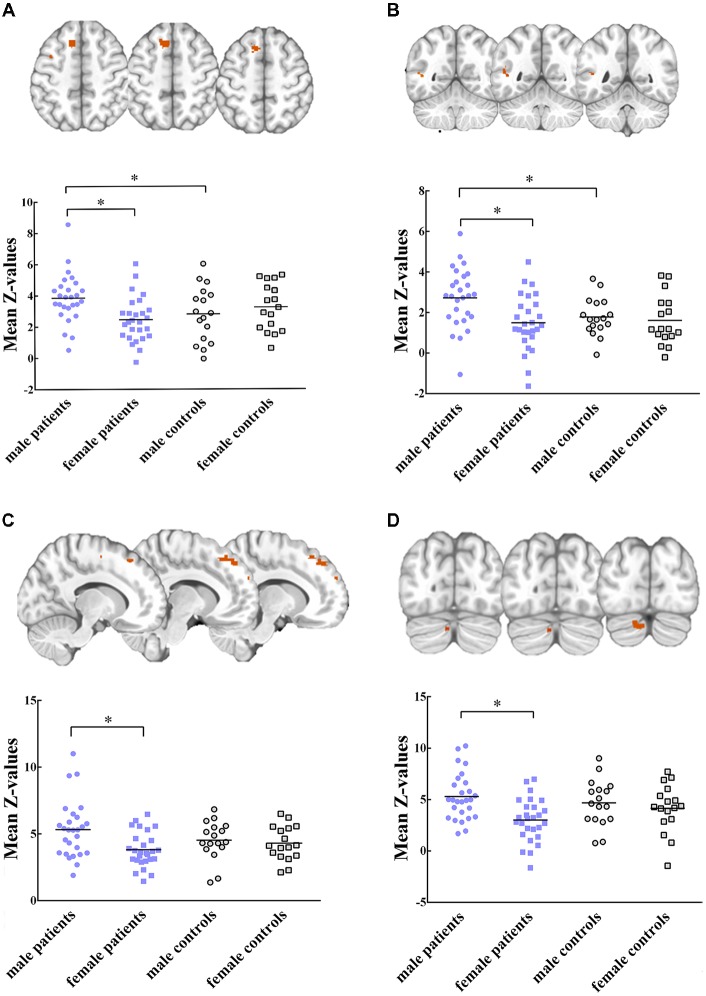
Intrinsic functional connectivity differences between male and female patients within **(A)** motor network, **(B)** ventral stream network, **(C)** executive function network **(D)** cerebellum are showed increased connectivity in male patients. Maps were thresholded at *P* < 0.05 (family wise error (FWE) corrected). The left side of the brain corresponds to the left side in the image. Scatterplots are displayed for regions-of-interest (ROI)-analysis among groups. Significant effects are denoted with asterisks between two groups, under *post hoc* restricted least significant difference (LSD) tests.

**Figure 3:**
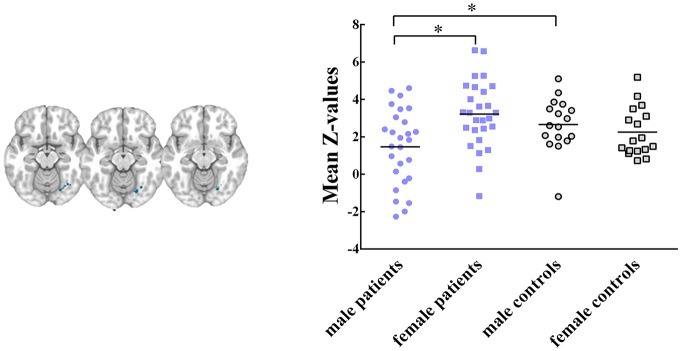
Intrinsic functional connectivity differences between male and female patients within visual network, where showed decreased connectivity in male patients. Maps were thresholded at *P* < 0.05 (FWE corrected). The left side of the brain corresponds to the left side in the image. Scatterplots are displayed for ROI-analysis among groups. Significant effects are denoted with asterisks between two groups, under *post hoc* restricted LSD tests.

Five RSNs showed significant voxel-wise differences in the spatial maps between male and female patients (*P* < 0.05, FWE corrected, Figures 2, 3). Compared with females, male patients showed increased intrinsic functional connectivity within the motor network (RSN 2), executive function network (RSN 3), ventral stream network (RSN 7), and cerebellum (RSN 8). By contrast, male patients performed lower connectivity than female patients in the visual network (RSN 12).

### ROI Data Analysis

Within these between-sex difference networks in patients, results of ANOVA demonstrated that intrinsic functional connectivity were significant different in clusters within the motor network (*P* = 0.012, *F*_(3,84)_ = 3.91), executive functional network (*P* = 0.011, *F*_(3, 84)_ = 3.96), ventral stream network (*P* = 0.004, *F*_(3,84)_ = 4.77), cerebellum (*P* = 0.003, *F*_(3,84)_ = 5.02) and visual network (*P* = 0.004, *F*_(3,84)_ = 4.88) among groups. *Post hoc* restricted least significant difference (LSD) tests of ANOVA further revealed that male patients had significant increased connectivity than male controls in the motor (RSN 2, *P* = 0.033) and ventral stream networks (RSN 7, *P* = 0.021). Male patients had lower connectivity strength in the visual network (RSN 12, *P* = 0.028) than male controls. No significant difference was found between female patients and female controls within these brain networks. In sum, the mild TBI effect on the sex difference was derived mainly from the male patients.

### The Relationship Between Abnormal Functional Connectivity and Clinical Performance

**Figure 4:**
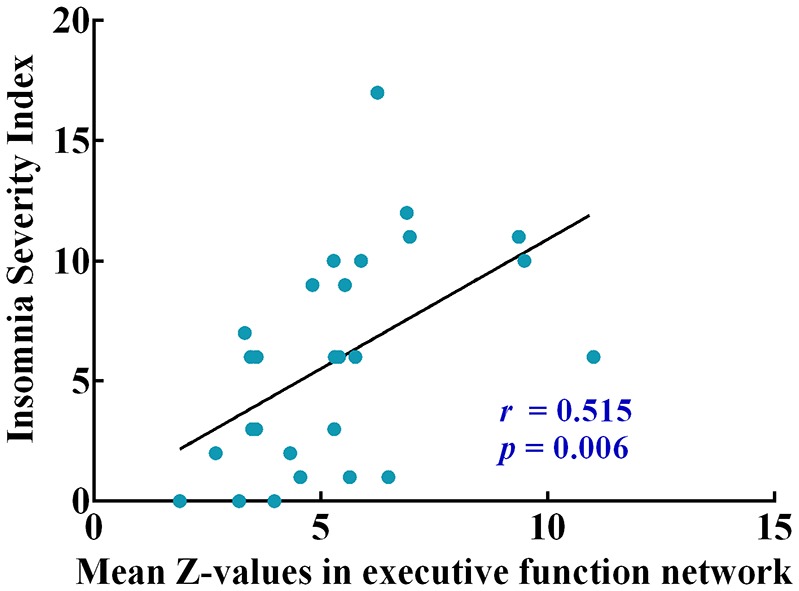
The pearson correlation coefficient plots correlating mean Z-values of intrinsic functional connectivity in executive function network with insomnia severity index (ISI).

Correlation analysis was then conducted and restricted into each brain network showing significant sex difference (RSNs 2, 3, 7, 8, 12) and clinical symptoms only in the male patients. Correlation analysis showed that functional connectivity within the executive function network (RSN 3) was positively correlated with ISI scores (*r* = 0.515, *P* = 0.006, Figure 4). A conservative corrected significance level of *P* < 0.0125 was considered following Bonferroni correction for the total number of clinical assessments involved (0.05/4).

## Discussion

Mild TBI is considered to induce abnormal resting state functional connectivity within intrinsic networks, however little is known about whether there is an effect of biological sex on response to TBI-related abnormalities (Shumskaya et al., 2012; Mayer et al., 2014). This study applied a whole-brain analysis to illustrate sex differences of resting-state functional connectivity from a network perspective after mild TBI. To the best of our knowledge, this is the first study to evaluate potential sex differences in the functional connectivity networks from a cohort of patients with acute mild TBI. The main findings of the present study are: (a) for the neuropsychological tests and self-report scales involved in the study, there were no significant differences between males and females either in mild TBI or control group; (b) however, male patients presented increased functional connectivity within motor, ventral steam, executive function and cerebellum networks, decreased connectivity within visual network compared with female patients; these identified RSNs have been previously associated with somatosensory and motor functions, executive control, self-related processing (Bressler and Menon, 2010); and (c) there was a positive correlation between functional connectivity within executive functional network and ISI scores in male patients.

No sex difference was observed in clinical scales after mild TBI in our study. Previous studies have attempted to discover sex effect on mild TBI using clinical cognitive outcomes (Covassin et al., 2012, 2013; Hsu et al., 2015). However, results of these experiments are controversial. Some have suggested that females report worse outcomes and more post-concussive symptoms compared with males following concussion (Broshek et al., 2005; Covassin et al., 2012; Cancelliere et al., 2016). One has found that women have superior executive functioning when compared with men after TBI (Niemeier et al., 2014). In consistent with several studies, our results had shown no substantial difference in outcome with regard to sex (Cantu et al., 2010; Frommer et al., 2011). Observational studies may be confounded by sociological pressures on males, leading to underreporting by male subjects (Fakhran et al., 2014). Thus, it is essential to develop a more objective measurement to evaluate the sex difference after injury.

In the present study, we selected seven functionally relevant RSNs. Between-group analysis reported the effects of sex difference on intrinsic functional connectivity within RSNs. Compared to female patients with mild TBI, male patients exhibited enhanced functional connectivity within the motor network (paracentral lobule, superior frontal gyrus, Figure 2A), ventral stream network (superior temporal gyrus, Figure 2B), executive function network (middle frontal gyrus, Figure 2C), and cerebellum (posterior lobe, Figure 2D). Male patients also presented higher connectivity than male controls in the motor and ventral steam network (Figures 2A,B). Motor network is involved in motor-learning, and it is suggested to have a potential role in disease-related functional connectivity alterations in motor tasks (Kumar et al., 2018). In a previous study, Shumskaya et al. (2012) find decreased functional connectivity within the motor-striatal network in the mild TBI group. Conversely, we observed increased connectivity in male patients when compared with either male HC or female patients. Nevertheless, there was no significant difference neither between female patients and female controls in the area, nor between male and female controls. We supposed that there might be physical male-specific stress response after acute injury. The ventral stream network is involved to the processing of visual information, as well as hearing (Tinelli et al., 2014; Dittinger et al., 2018). Our results showed significantly increased functional connectivity in the superior temporal gyrus in male patients. In executive function network, male patients showed greater connectivity than female patients on the middle frontal gyrus. A previous study suggest that increased connectivity found in the mild TBI group might lead to the increase of awareness from the external environment during the acute stage, and it might explain cognitive over-fatigue recounted by patients with mild TBI (Shumskaya et al., 2012). Similarly, damaged self-awareness has also been attested previously in patients with TBI with frontal lobe injury (Spikman and van der Naalt, 2010). Within cerebellum network, comparing to female patients, male patients showed increased connectivity on the anterior lobe and posterior lobe. However, few researches have investigated functional connectivity in cerebellum, since there is no region of interest in studies. A research about sex differences on cerebellum should be done in future. In brief, the hyper-connectivity in male patients indicated that men had a different strategy in information processing when suffering concussion.

Further, male patients showed significant decreased functional connectivity on occipital lobe, which considered to be one component of visual network (Figure 3), compared with female patients and male controls. The visual network is related to the processing of visual information, and visual dysfunctions in mild TBI include photosensitivity or photaesthesia, double vision and vision deficits (Goodrich et al., 2007; Cockerham et al., 2009). Visual dysfunction had been found to be associated with high level processing defects, such as the speed of understanding and reading (Capó-Aponte et al., 2012). Mild TBI may destroy the transmission of information, and then affect perception, cognition and behavior. In visual network, female patients showed no significant difference than female controls. We suspected that males had weaker visual adjustment ability with compensation than females following injury. For the acute mild TBI, females may have the ability to protect their visual network with production of some regulatory substance.

Moreover, a positive correlation between functional connectivity within executive function network and ISI scores was discovered in male patients, suggesting the hyperconnectivity in executive function network was detrimental to male’s sleeping after mild TBI. One study has reported that the hyperconnectivity with dorsal medial prefrontal cortex (dlPFC) across the executive function network may explain impaired concentration, increased rumination and self-focus in major depressive disorder (Crowther et al., 2015). It has been demonstrated that a single night of sleep deprivation is harmful to cognitive abilities, ranging from phasic alertness to executive functions (Harrison et al., 2000; Doran et al., 2001; Muto et al., 2012). The fragmented sleep can also make impairments on hyper-arousal and executive dysfunction (Stoffers et al., 2014). The results in our study may indicate that males were more vulnerable than females when suffering mild TBI.

Several limitations of our present study should be noted. First, it cannot infer direct causal effects of sex differences from current results in the study. Sex differences on functional connectivity are the result of interaction between genetics and external environmental factors (Kaczkurkin et al., 2016). Nonetheless, in specific RSNs, the significant sex differences presented on functional connectivity suggest particular adjustment for males following mild TBI, and may represent an important mechanism. Second, current study about mild TBI is limited to the acute period. Long-term differences of functional connectivity or prognosis between male and female patients cannot be accomplished. Recent research had shown that the strength of these network connections would be increased or decreased in a rapid and reversible manner to achieve a dynamic network connection (DNC; Arnsten et al., 2010). We need a longitudinal follow-up instead of once time point study in future. Third, the hormone status, which has been shown to affect neuropsychologic data and image status, is not unified (Bhagia et al., 2010; Xu et al., 2010; McAllister et al., 2011; Tamargo et al., 2017; Yamakawa et al., 2017). The study variables cannot be completely controlled. Furthermore, a combination of imaging methods, such as SWI, diffusion tensor imaging for integrity of white matter cellulose, MR perfusion study and MR spectroscopy for metabolite study should be compared, which may help to understand the structural pathophysiology of mild TBI and the causes of sex differences, to draw a more statistically significant conclusion.

## Conclusion

In summary, the current findings of our research confirmed that there were significant sex differences on functional connectivity within specific RSNs following mild TBI. Male patients showed hyper-connectivity than female patients in four of selected RSNs, including motor, ventral stream, executive function and cerebellum networks. Hypo-connectivity in male patients than female patients was found in visual network, suggesting a king of female compensation mechanism. We found that the abnormality of the functional connectivity of RSNs in acute mild TBI showed the possibility of brain recombination after trauma, mainly concerning male-specific.

## Author Contributions

SW and LH performed the experiment, analyzed image data and drafted the manuscript. JC, WH, CS, ZW, SG and XN performed the experiment and statistical results. DDZ, CG, GB, LY, DBZ and NZ collected the data involved in the study. BY, MZ and LB designed the study and gave critical comments on the manuscript.

## Conflict of Interest Statement

The authors declare that the research was conducted in the absence of any commercial or financial relationships that could be construed as a potential conflict of interest.
